# Fabrication and characterization of well-aligned plasmonic nanopillars with ultrasmall separations

**DOI:** 10.1186/1556-276X-9-299

**Published:** 2014-06-13

**Authors:** Guangyuan Si, Xiaoxiao Jiang, Jiangtao Lv, Qiongchan Gu, Fengwen Wang

**Affiliations:** 1 College of Information Science and Engineering, Northeastern University, 110004 Shenyang, China

**Keywords:** Plasmonic, Nanopillars, Dense arrays

## Abstract

We show the fabrication of well-aligned gold and silver nanopillars with various array parameters via interference lithography followed by ion beam milling and compare the etching rates of these two metallic materials. Silver is suitable for fabricating ultrafine arrays with ultrasmall separations due to high milling rates. The optical properties of the fabricated nanopillars are specifically characterized from both normal incidence and oblique incident angles. Tunable surface plasmon resonances are achieved with varying structural parameters. Strong coupling effects are enabled when the separation between adjacent nanopillars is dramatically reduced, leading to useful applications in sensing and waveguiding.

## Background

Known as the electromagnetic waves propagating along metal-dielectric interfaces, surface plasmons (SPs) have drawn increasing attention in recent years [1-5]. Many plasmon-enabled applications have been developed due to their unique optical properties and particular ability of manipulating light at the nanometer scale. Additionally, SP-based waveguides are useful for developing devices with ultrahigh sensitivity and figure of merit because the near-field of electromagnetic waves can be significantly enhanced using different plasmonic nanostructures. Various plasmonic nanostructures, including nanopillars for waveguiding [6-8], and bio-sensing [9-11], or photonic crystals for efficient cavity coupling [12], have been demonstrated recently. Moreover, extensive useful applications have been triggered by plasmonics in super-resolution imaging [13-15], cloaking [16-18], energy harvesting [19-21], and color filtering [22-25]. Various applications (plasmonic absorbers, for instance) have been reported by using nanodisks [26-28] or nanopillars [29] to modify the surface profile, allowing for tight confinement of more energy inside the functional layer of a solar cell. Such nanodisks/nanopillars that act as plasmonic absorbers (also known as plasmonic blackbodies) are extremely useful for energy harvesting. Metal nanopillars or wires excited by electromagnetic waves show resonance characteristics which are highly dependent on geometric parameters. In the optical regime, metals are dispersive materials with finite conductivity. Either surface plasmon polaritons (SPPs) or localized surface plasmon resonances (LSPRs) reveal salient resonance features, and the optical properties of metal nanopillars are mainly determined by their shape, size, and even the dielectric environment. Recently, the fascinating optical properties of small nanopillars/particles [30-34] and other different geometries [35-40] have been extensively investigated both experimentally and theoretically, providing a new pathway for manipulating light at the subwavelength scale.

Due to important advances in nanofabrication techniques, plasmonic nanostructures and related devices are presently gaining tremendous technological significance in nanophotonics and optics. Nanostructures could provide intriguing possibilities for resolving those challenges and improving device performance. Well-aligned nanopillars with perpendicular orientations to the substrate are becoming the main building blocks for new optical devices with promising potential applications [41]. Here we explore, experimentally and theoretically, the optical properties of periodic nanopillars perpendicularly aligned on the supporting substrate. Combination of interference lithography (IL) and ion beam milling (IBM) techniques enables scalable fabrication of such nanopillars with excellent dimensional control and high uniformity. Detailed experimental results show that silver (Ag) has a much higher etching rate than gold (Au) under the same milling conditions, making Ag a perfect candidate for the construction of plasmonic ultrasmall features. In addition, nanopillar arrays with ultrasmall inter-pillar separations are fabricated and optically characterized.

## Methods

Quartz substrates were first cleaned with acetone in an ultrasonic bath followed by isopropyl alcohol (IPA) and deionized water washing and finally blow-dried with a nitrogen gun. Subsequently, Au or Ag films with different thicknesses were deposited on quartz substrates with 4-nm titanium as the adhesion layer by electron beam evaporation (Auto 306, Edwards, Crawley, UK) at a base pressure of about 3 × 10^-7^ mbar. In order to minimize the deposition-introduced roughness, low evaporation rates were applied (less than 0.03 nm/s). Afterwards, positive resist (S1805, Dow, Midland, MI, USA) was used to define nanopillar arrays on the metal (Au or Ag) layer supported by a quartz substrate (refractive index = 1.46) with a laser holography system using a 325-nm helium-cadmium laser, serving as the IBM mask after development.

**Figure 1:**
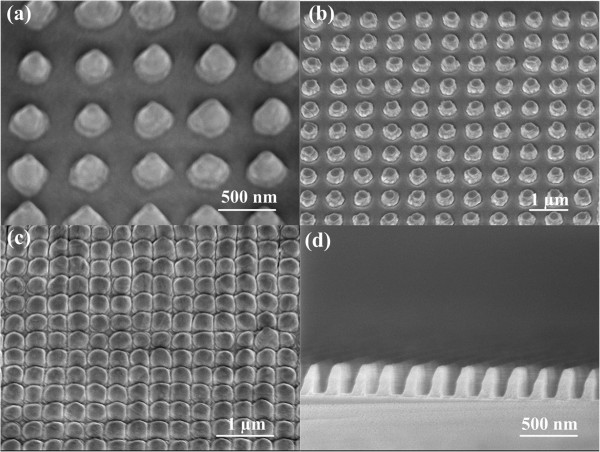
**SEM images of nanopillars with different outlines and profiles. (a)** Cone-shaped particles. **(b)** Normal nanopillars. **(c)** Nanopillars with ultrasmall separations. **(d)** Cross-sectional view of pagoda-shaped nanopillars. Note that the materials used in **(a)** and **(b)** and in **(c)** and **(d)** are Au and Ag, respectively.

During the IBM process (Microetch 1201, Veeco Instruments, Plainview, NY, USA), argon was ionized and accelerated in an electric field to a high energy level. Argon ions struck the target materials while the sample plate rotated, ensuring homogeneous removal of waste material and straight sidewalls in all features with nearly zero undercutting. The work plate was cooled and tilted 10° to the normal of the incident beam to ensure even uniformity of the ion bombardment. At last, resist residue was removed by Microposit Remover 1165 (Rohm and Haas, Philadelphia, PA, USA) and cleaned up with IPA and deionized water. Detailed milling parameters are summarized in Table 1. The measured milling rate for Au and Ag is 23 and 61 nm/min, respectively.Compared with other fabrication methods, IL has idiographic advantages. For instance, IL allows for processing a complete substrate with one single exposure or several times of full-area exposures to define complex patterns. More importantly, IL can offer the possibility to construct homogeneous micro- or nanometer-structured surfaces on areas with wafer scale that is either impossible or extremely time consuming with other patterning techniques. In addition, one can precisely control the geometry of the arrays in a wide range by changing the processing parameters such as the incident angle and exposure time. As shown in Figure 1, nanopillars with varying profiles are achieved by accurately controlling the milling conditions. One can clearly observe cone-shaped particles in Figure 1a, which were achieved by oblique milling. In Figure 1b, normal round-shaped nanopillars are shown. Rough fringes are caused by redeposition which is almost inevitable in all ion-involved milling processes. Further, Figure 1c demonstrates nanopillars with ultrasmall separations. Note that the round shapes are replaced by quadrate outlines since the individual nanopillars are approaching each other. Smallest features of approximately 10 nm are realized. Figure 1d shows the cross sections of pagoda nanopillars with high aspect ratios (100-nm average diameter and 270-nm height).

**Table 1:** Parameters summary for the IBM process in this work

**Parameter**	**Value**	**Unit**
Voltage	300	V
Current	200	mA
Suppressor	150	V
Discharge	60	V
Magnet current	485	mA
Flow rate	30	sccm

The optical properties of the fabricated nanopillars under normal incidence were measured using a commercial system (UV-VIS-NIR microspectrophotometer QDI 2010™, CRAIC Technologies, Inc., San Dimas, CA, USA). A × 36 objective lens with the numerical aperture of 0.5 was employed with a 75-W xenon lamp which provided a broadband spectrum. Using a beam splitter, the partial power of the incident light beam was focused onto the sample surface through the objective lens. The spectrum acquisition for all measurements was performed with a sampling aperture size of 7.1 × 7.1 μm^2^. Transmission and reflection were measured with respect to the light through a bare quartz substrate and an aluminum mirror, respectively. To characterize the optical properties from oblique angles, an ellipsometry setup (Uvisel, Horiba Jobin Yvon, Kyoto, Japan) was employed with a broadband light source.

## Results and discussion

**Figure 2:**
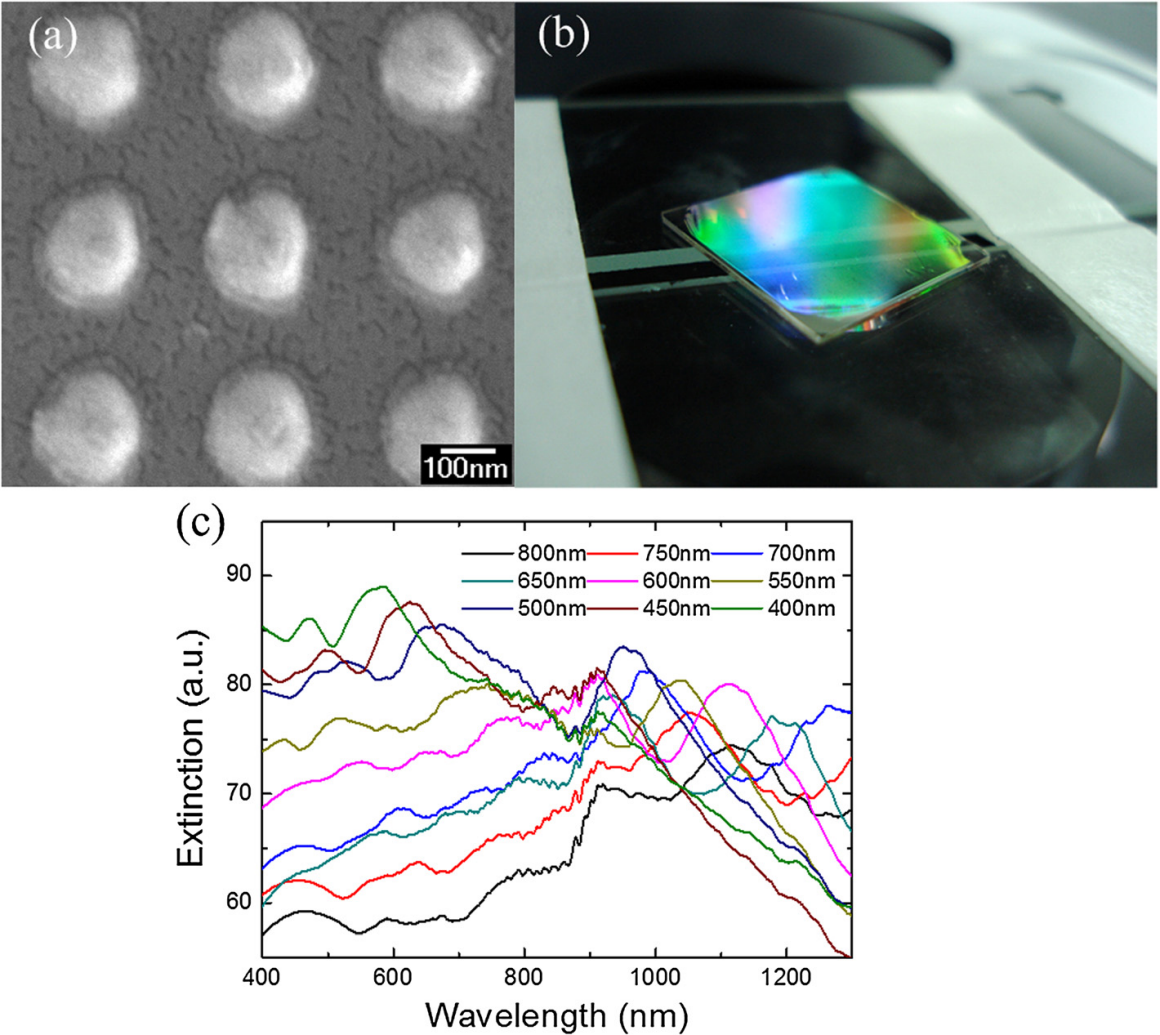
**SEM image, optical image, and extinction spectra of Ag nanopillars. (a)** Top-view SEM image of Ag nanopillars with 400-nm periodicity. **(b)** Optical image of nanopillars supported by a quartz substrate. **(c)** Measured extinction spectra for nanopillar arrays with varying periodicities.

Figure 2a demonstrates the scanning electron microscopy (SEM) image of the top view of the fabricated Ag nanopillars with 400-nm periodicity. As can be seen, the fringe of the nanopillars presents a brighter color than the other areas due to different contrast which is caused by materials redeposition during milling. Figure 2b is the optical image of nanopillars supported by a quartz substrate with the size of 1.5 × 1.5 cm^2^. The corners show defects caused by fabrication imperfections since the pattern area is limited during holography and uneven distribution of resist during spin coating. The extinction spectra for nanopillar arrays with varying periodicities are plotted in Figure 2c. One can clearly observe tunable LSPRs and redshift of resonance peaks with increasing periodicities. Besides, relatively large full width at half maximum can be seen for resonance peaks after 900 nm.

**Figure 3:**
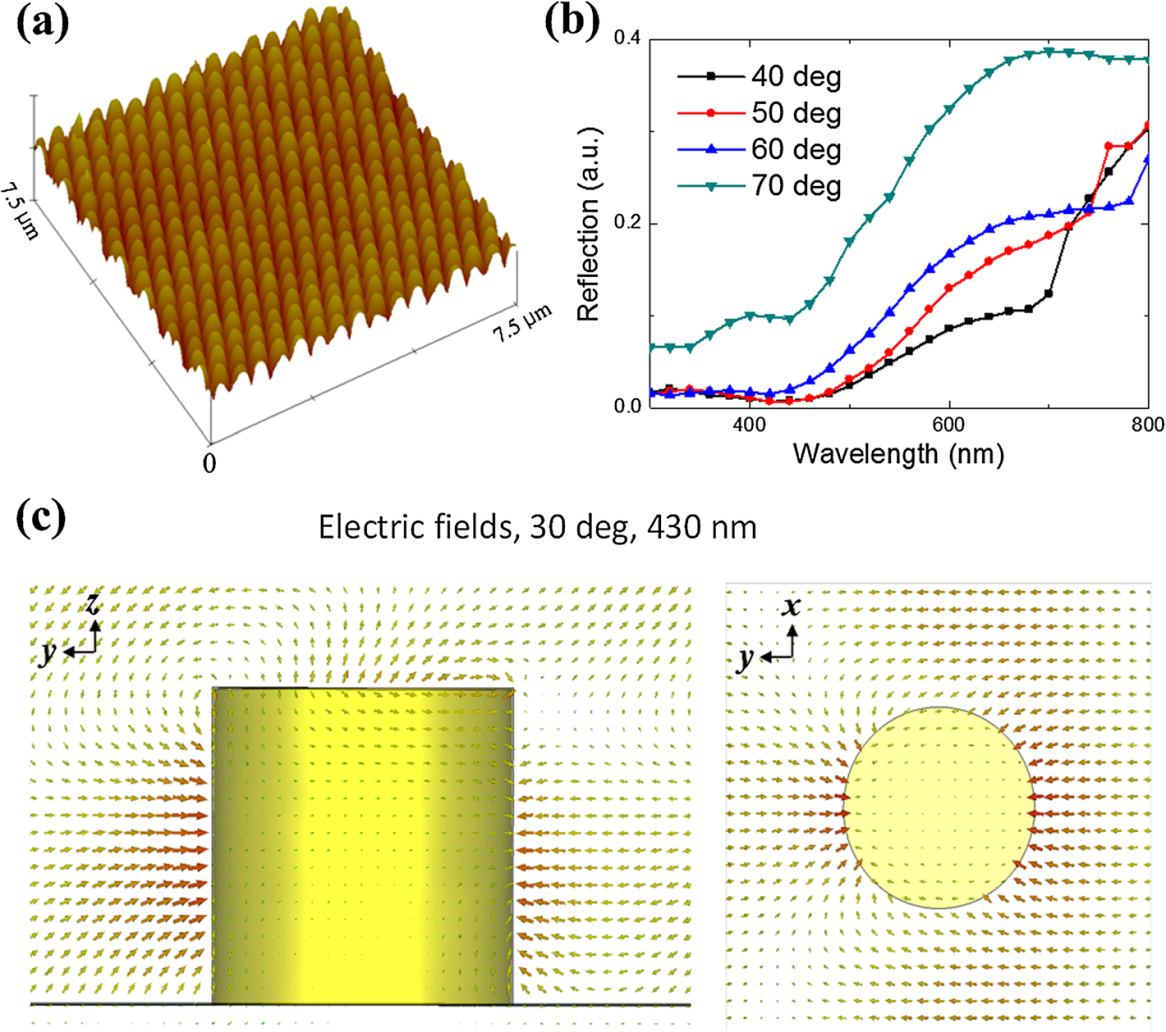
**AFM image, reflectance, and electric field distributions of Au nanopillars. (a)** AFM image of Au nanopillars with 450-nm periodicity. **(b)** Measured reflectance of Au nanopillar arrays with varying incident angles. **(c)** Calculated side-view (left) and top-view (right) electric field distributions of a nanopillar at 30° incidence at the wavelength of 430 nm.

**Figure 4:**
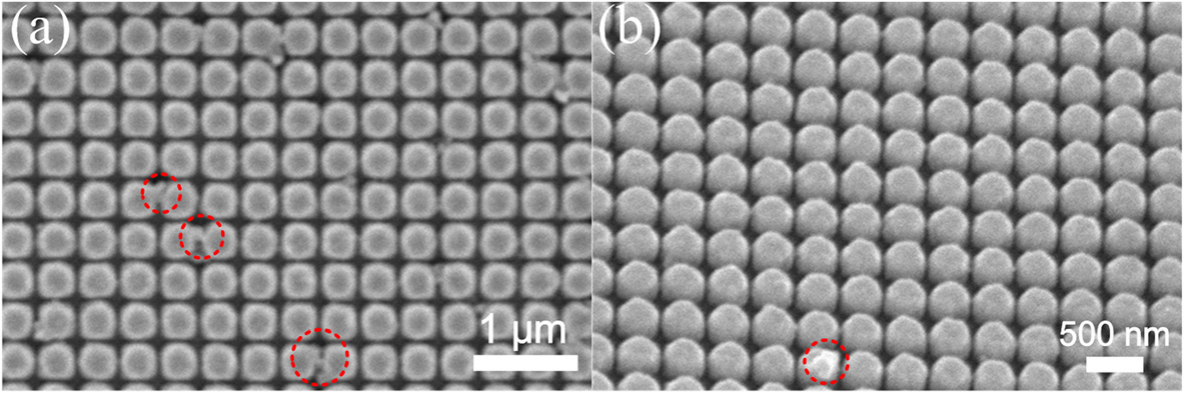
**Top-view (a) and oblique-view (b) SEM images of Ag nanopillar arrays with ultrasmall separations.** Typical fabrication imperfections are indicated with red circles which are almost inevitable in the milling process.

**Figure 5:**
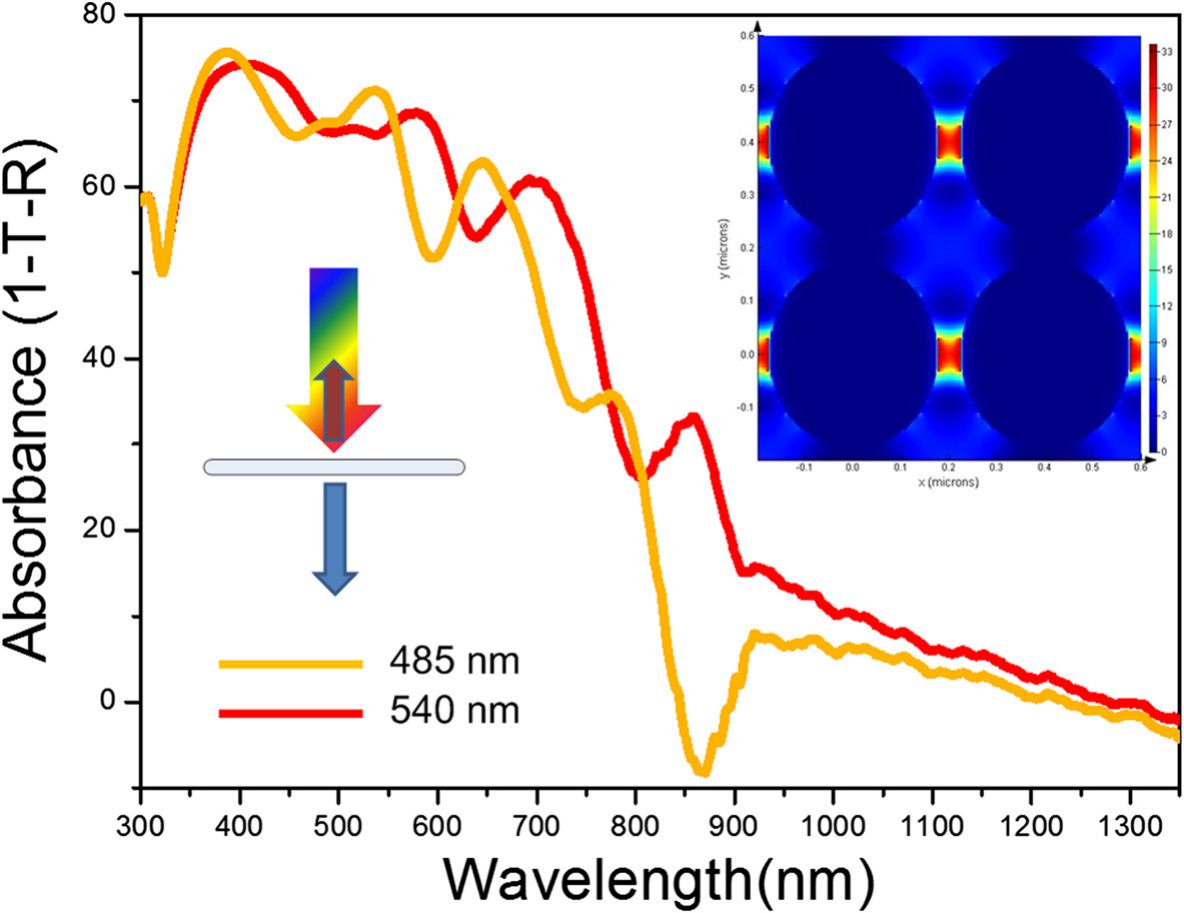
**Measured absorbance of Ag nanopillar arrays with 485- and 540-nm periodicities and 35- and 40-nm inter-pillar separations.** The insets show the schematic diagram for experimental characterization at normal incidence and the electric field distribution at plasmon resonance.

Figure 3a shows the atomic force microscopy (AFM) image of the Au nanopillar array with 450-nm periodicity. As can be seen, nanopillars with uniform shapes are achieved. The measured reflectance spectra of nanopillar arrays with different incident angles (40° to 70° in 10° increments) as a function of wavelength are plotted in Figure 3b. Tunable plasmon resonance with varying incident angles can be observed. Figure 3c shows the electric near-field distribution of a single nanopillar at 30° to the incidence normal at the wavelength of 430 nm calculated by using CST microwave studio. During simulations, one unit cell was considered which consisted of a vertically oriented cylindrical Au nanopillar. Periodic boundary conditions were assigned to the lateral walls and Floquet ports were imposed on top and bottom of the unit cell to mimic an infinite periodic array with a periodicity of *p* = 450 nm. The nanopillar has a radius of *r* = 100 nm and a height of *h* = 200 nm. A fifth-order Drude-Lorentz model was employed to fit the measured permittivity of Au [42]. It is observed that at the wavelength corresponding to the peak of specular reflection for each angle of incidence case, the electric field exhibits curl-like patterns, concentrating near the vertical surface of the nanopillar.As mentioned above, Ag has a much higher etching rate than Au under the same milling parameters using ion beams. Therefore, Ag has a larger selectivity than Au with the same resist mask (fixed thickness) for milling. Figure 4a,b shows the top-view and oblique-view SEM images of Ag nanopillar arrays with ultrasmall gap sizes, respectively. The average measured smallest gap width is approximately 10 nm. Dome-shaped profiles can be observed from Figure 4b, which is mainly caused by materials redeposition during the milling process. Note that the gaps between neighboring nanopillars have been milled through to the surface of the substrate. Typical fabrication imperfections are highlighted with red circles.The measured absorbance spectra for two Ag nanopillar arrays with different periodicities and ultrasmall inter-pillar separations are plotted in Figure 5. The LSPRs in nanopillars can be described as a series of longitudinal standing waves with an increasing number of harmonics at shorter wavelengths. In addition, the LSPRs are laterally confined and bounded between adjacent nanopillars. The spectra also show the effect of periodicity variation and reveal different regimes. Very little radiative coupling occurs when the diffraction edge is on the high-energy side of the main LSPR since the allowed diffracted orders have higher energy than the plasmon resonance. Most of the LSPRs confined within the nanopillar array exist as higher-order modes. Note that the standing waves within the nanopillars can be influenced by the coupling of transverse plasmon modes between nanopillars, leading to different resonances described for separate nanopillars. Additionally, Fano-type line shapes are observed which result from the interference between directly transmitted and scattered energy. Such nanopillars have great potential for sensing purposes due to significantly enhanced near-field intensity which can be clearly observed from the inset of Figure 5, possessing the key advantage of plasmonic-based sensors which may enable new opportunities for sensing geometries and strategies.

## Conclusions

To conclude, we have demonstrated the fabrication of well-aligned plasmonic nanopillars by combining IL and IBM techniques. Using arrays with different geometric parameters, tunable plasmon resonances are simply achieved. It is found that Ag has a much higher milling rate than Au under the same experimental conditions, which makes Ag suitable for constructing fine nanostructures with ultrasmall features and high aspect ratios. The optical properties of the fabricated nanopillars are characterized both experimentally and theoretically. The approach developed in this work may trigger new applications in plasmon-assisted sensing and detecting.

## Abbreviations

AFM: atomic force microscopy; IBM: ion beam milling; IL: interference lithography; IPA: isopropyl alcohol; LSPRs: localized surface plasmon resonances; SEM: scanning electron microscopy; SPs: surface plasmons; SPPs: surface plasmon polaritons.

## Competing interests

The authors declare that they have no competing interests.

## Authors’ contributions

GS and XJ fabricated and measured the nanopillars. QG and JL helped with the simulations. FW supervised the project. All authors read and approved the final manuscript.

## References

[B1] Ebbesen TW Lezec HJ Ghaemi HF Thio T Wolff PA Extraordinary optical transmission through sub-wavelength hole arrays Nature 1998 9 667 669 10.1038/35570

[B2] Liu YJ Zheng YB Liou J Chiang IK Khoo IC Huang TJ All-optical modulation of localized surface plasmon coupling in a hybrid system composed of photo-switchable gratings and Au nanodisk arrays J Phys Chem C 2011 9 7717 7722 10.1021/jp111256u PMC3105912 21643480

[B3] Zhao Y Nawaz AA Lin SS Hao Q Kiraly B Huang TJ Nanoscale super-resolution imaging via metal-dielectric metamaterial lens system J Phys D Appl Phys 2011 9 41501

[B4] Liu YJ Hao QZ Smalley JST Liou J Khoo IC Huang TJ A frequency-addressed plasmonic switch based on dual-frequency liquid crystals Appl Phys Lett 2010 9 091101 10.1063/1.3483156

[B5] Zhao Y Lin SS Nawaz AA Kiraly B Hao Q Liu Y Huang TJ Beam bending via plasmonic lenses Opt Express 2010 9 23458 23465 10.1364/OE.18.023458 21164688

[B6] Gao H Liu C Jeong HE Yang P Plasmon-enhanced photocatalytic activity of iron oxide on gold nanopillars ACS Nano 2012 9 234 240 10.1021/nn203457a 22147636

[B7] Zhang J Cai L Bai W Song G Hybrid waveguide-plasmon resonances in gold pillar arrays on top of a dielectric waveguide Opt Lett 2010 9 3408 3410 10.1364/OL.35.003408 20967082

[B8] Wang K Crozier KB Plasmonic trapping with a gold nanopillar ChemPhysChem 2012 9 2639 2648 10.1002/cphc.201200121 22623501

[B9] Cetin AE Yanik AA Yilmaz C Somu S Busnaina A Altug H Monopole antenna arrays for optical trapping, spectroscopy, and sensing Appl Phys Lett 2011 9 111110 10.1063/1.3559620

[B10] Kubo W Fujikawa S Au double nanopillars with nanogap for plasmonic sensor Nano Lett 2011 9 8 15 10.1021/nl100787b 21114297

[B11] Kabashin AV Evans P Pastkovsky S Hendren W Wurtz GA Atkinson R Pollard R Podolskiy VA Zayats AV Plasmonic nanorod metamaterials for biosensing Nat Mater 2009 9 867 871 10.1038/nmat2546 19820701

[B12] Chigrin D Lavrinenko A Torres CS Numerical characterization of nanopillar photonic crystal waveguides and directional couplers Opt Quant Electron 2005 9 331 341 10.1007/s11082-005-1189-1

[B13] Zhao Y Gan D Cui J Wang C Du C Luo X Super resolution imaging by compensating oblique lens with metallodielectric films Opt Express 2008 9 5697 5707 10.1364/OE.16.005697 18542677

[B14] Melville DOS Blaikie RJ Super-resolution imaging through a planar silver layer Opt Express 2005 9 2127 2134 10.1364/OPEX.13.002127 19495100

[B15] Casse BDF Lu WT Huang YJ Gultepe E Menon L Sridhar S Super-resolution imaging using a three-dimensional metamaterials nanolens Appl Phys Lett 2010 9 023114 10.1063/1.3291677

[B16] Cao T Wang S Topological insulator metamaterials with tunable negative refractive index in the optical region Nanoscale Res Lett 2013 9 526 10.1186/1556-276X-8-526 24330596 PMC3866564

[B17] Cai W Chettiar UK Kildishev AV Shalaev VM Optical cloaking with metamaterials Nat Photon 2007 9 224 227 10.1038/nphoton.2007.28

[B18] Chen H Chan CT Acoustic cloaking in three dimensions using acoustic metamaterials Appl Phys Lett 2007 9 183518 10.1063/1.2803315

[B19] Xue J Zhu Q Liu J Li Y Zhou ZK Lin Z Yan J Li J Wang XH Gold nanoarray deposited using alternating current for emission rate-manipulating nanoantenna Nanoscale Res Lett 2013 9 295 10.1186/1556-276X-8-295 23799880 PMC3694511

[B20] Aubry A Lei DY Fernández-Domínguez AI Sonnefraud Y Maier SA Pendry JB Plasmonic light-harvesting devices over the whole visible spectrum Nano Lett 2010 9 2574 2579 10.1021/nl101235d 20518545

[B21] Cole JR Halas NJ Optimized plasmonic nanoparticle distributions for solar spectrum harvesting Appl Phys Lett 2006 9 153120 10.1063/1.2360918

[B22] Si G Zhao Y Liu H Teo S Zhang M Huang TJ Danner AJ Teng JH Annular aperture array based color filter Appl Phys Lett 2011 9 033105 10.1063/1.3608147

[B23] Liu YJ Si GY Leong ESP Xiang N Danner AJ Teng JH Light-driven plasmonic color filters by overlaying photoresponsive liquid crystals on gold annular aperture arrays Adv Mater 2012 9 OP131 OP135 22438069 10.1002/adma.201104440

[B24] Si G Zhao Y Lv J Lu M Wang F Liu H Xiang N Huang TJ Danner AJ Teng J Liu YJ Reflective plasmonic color filters based on lithographically patterned silver nanorod arrays Nanoscale 2013 9 6243 6248 10.1039/c3nr01419c 23685642

[B25] Si G Zhao Y Leong ESP Liu YJ Liquid-crystal-enabled active plasmonics: a review Materials 2014 9 1296 1317 10.3390/ma7021296 PMC5453087 28788515

[B26] Zhao Y Hao Q Ma Y Lu M Zhang B Lapsley M Khoo IC Huang TJ Light-driven tunable dual-band plasmonic absorber using liquid-crystal-coated asymmetric nanodisk array Appl Phys Lett 2012 9 053119 10.1063/1.3681808

[B27] Zhang B Zhao Y Hao Q Kiraly B Khoo IC Chen S Huang TJ Polarization independent dual-band infrared perfect absorber based on a metal-dielectric-metal elliptical nanodisk array Opt Express 2011 9 15221 15228 10.1364/OE.19.015221 21934885

[B28] Liu N Mesch M Weiss T Hentschel M Giessen H Infrared perfect absorber and its application as plasmonic sensor Nano Lett 2010 9 2342 2348 10.1021/nl9041033 20560590

[B29] Fan Z Kapadia R Leu PW Zhang X Chueh YL Takei K Yu K Jamshidi A Rathore AA Ruebusch DJ Wu M Javey A Ordered arrays of dual-diameter nanopillars for maximized optical absorption Nano Lett 2010 9 3823 3827 10.1021/nl1010788 20491498

[B30] Caldwell JD Glembocki O Bezares FJ Bassim ND Rendell RW Feygelson M Ukaegbu M Kasica R Shirey L Hosten C Plasmonic nanopillar arrays for large-area, high-enhancement surface-enhanced Raman scattering sensors ACS Nano 2011 9 4046 4055 10.1021/nn200636t 21480637

[B31] Senanayake P Hung CH Shapiro J Scofield A Lin A Williams BS Huffaker DL 3D nanopillar optical antenna photodetectors Opt Express 2012 9 25489 25496 10.1364/OE.20.025489 23187366

[B32] Caldwell JD Glembocki O Bezares FJ Kariniemi MI Niinisto JT Hatanpaa TT Rendell RW Ukaegbu M Ritala MK Prokes SM Hosten CM Leskela MA Kasica R Large-area plasmonic hot-spot arrays: sub-2 nm interparticle separations with plasma-enhanced atomic layer deposition of Ag on periodic arrays of Si nanopillars Opt Express 2011 9 26056 26064 10.1364/OE.19.026056 22274194

[B33] Tsai SJ Ballarotto M Romero DB Herman WN Kan HC Phaneuf RJ Effect of gold nanopillar arrays on the absorption spectrum of a bulk heterojunction organic solar cell Opt Express 2010 9 A528 A535 10.1364/OE.18.00A528 21165085

[B34] Lin HY Kuo Y Liao CY Yang CC Kiang YW Surface plasmon effects in the absorption enhancements of amorphous silicon solar cells with periodical metal nanowall and nanopillar structures Opt Express 2012 9 A104 A118 10.1364/OE.20.00A104 22379680

[B35] Zeng B Gao Y Bartoli FJ Ultrathin nanostructured metals for highly transmissive plasmonic subtractive color filters Sci Rep 2013 9 2840 24100869 10.1038/srep02840 PMC3792416

[B36] Zeng B Yang X Wang C Luo X Plasmonic interference nanolithography with a double-layer planar silver lens structure Opt Express 2009 9 16783 16791 10.1364/OE.17.016783 19770895

[B37] Zeng B Gan Q Kafafi ZH Bartoli FJ Polymeric photovoltaics with various metallic plasmonic nanostructures J Appl Phys 2013 9 063109 10.1063/1.4790504

[B38] Zeng B Yang X Wang C Feng Q Luo X Super-resolution imaging at different wavelengths by using a one-dimensional metamaterial structure J Opt 2010 9 035104 10.1088/2040-8978/12/3/035104

[B39] Gao Y Xin Z Zeng B Gan Q Cheng X Bartoli FJ Plasmonic interferometric sensor arrays for high-performance label-free biomolecular detection Lab Chip 2013 9 4755 4764 10.1039/c3lc50863c 24173621

[B40] Xu T Fang L Zeng B Liu Y Wang C Feng Q Luo X Subwavelength nanolithography based on unidirectional excitation of surface plasmons J Opt A Pure Appl Opt 2009 9 085003 10.1088/1464-4258/11/8/085003

[B41] Drezet A Koller D Hohenau A Leitner A Aussenegg FR Krenn JR Plasmonic crystal demultiplexer and multiports Nano Lett 2007 9 1697 1700 10.1021/nl070682p 17500579

[B42] Johnson PB Christy RW Optical constants of the noble metals Phys Rev B 1972 9 4370 4379 10.1103/PhysRevB.6.4370

